# Testing the Safety of Piriformis Dry Needling Interventions: An Observational Study Evaluating the Predictive Value of Anthropometric and Demographic Factors

**DOI:** 10.3390/jcm13226674

**Published:** 2024-11-07

**Authors:** Juan Antonio Valera-Calero, Umut Varol, Gustavo Plaza-Manzano, César Fernández-de-las-Peñas, Pedro Belón-Pérez, Mónica López-Redondo, Marcos José Navarro-Santana

**Affiliations:** 1 Department of Radiology, Rehabilitation and Physiotherapy, Faculty of Nursery, Physiotherapy and Podiatry, Universidad Complutense Madrid, 28040 Madrid, Spain; juavaler@ucm.es (J.A.V.-C.); gusplaza@ucm.es (G.P.-M.); marconav@ucm.es (M.J.N.-S.); 2 Grupo InPhysio, Instituto de Investigación Sanitaria del Hospital Clínico San Carlos (IdISSC), 28040 Madrid, Spain; 3 Escuela Internacional de Doctorado, Universidad Rey Juan Carlos, 28922 Alcorcón, Spain; au.varol.2022@alumnos.urjc.es; 4 Department of Physical Therapy, Occupational Therapy, Rehabilitation and Physical Medicine, Universidad Rey Juan Carlos, 28922 Alcorcón, Spain; cesar.fernandez@urjc.es; 5 Cátedra Institucional en Docencia, Clínica e Investigación en Fisioterapia: Terapia Manual, Punción Seca y Ejercicio Terapéutico, Universidad Rey Juan Carlos, 28922 Alcorcón, Spain; 6 Department of Physical Therapy, Real Madrid C.F., 28055 Madrid, Spain; pebelon@gmail.com; 7 Faculty of Health Sciences, Universidad Francisco de Vitoria, 28223 Madrid, Spain

**Keywords:** accident prevention, adverse events, dry needling, medical decision making, piriformis muscle

## Abstract

**Objectives**: The dry needling of the piriformis muscle (especially in the medial region) is a challenging procedure since there is a high risk of accidentally puncturing the sciatic nerve. This study aimed to explain the variance of the deep limit of the piriformis based on anthropometric and demographic predictors potentially associated with it by exploring if clinicians can select the optimal needle length needed accurately to avoid accidental punctures of the sciatic nerve during palpation-guided dry needling interventions. **Methods**: An observational study was conducted that included fifty-six patients with piriformis muscle syndrome. We recorded the skin-to-sciatic nerve distance at the location with greatest risk of accidental sciatic puncture (assessed with ultrasound imaging) and demographic (e.g., age, gender, height, weight and body mass index—BMI) and anthropometric (hip circumference) variables. **Results**: Thirty-four males (*n* = 34) and twenty-two females (*n* = 22) were analyzed. Although men presented a significantly greater hip circumference than women (*p* = 0.007), no skin-to-sciatic nerve distance differences were observed (*p* > 0.05). Correlation analyses revealed that the sciatic nerve’s depth is associated with weight, BMI and hip perimeter (all, *p* < 0.01) but not with age or height (*p* > 0.05). Due to shared variance and multicollinearity, the hip circumference was the only predictor included in the regression model, explaining 37.9% of the piriformis muscle’s deeper fascia depth variance (R^2^ Adjusted = 0.379). **Conclusions**: Although the use of landmarks and measuring the hip perimeter may result in greater dry needling accuracy and a lower risk of adverse events derived from accidental sciatic nerve puncture, ultrasound guidance is encouraged as is the safest method for avoiding serious adverse events.

## 1. Introduction

The piriformis muscle is a flat triangular-shaped muscle located in the gluteal region. This muscle originates from the anterior surface of the lateral sacrum process, passes from the pelvis to the gluteal region through the greater sciatic notch and inserts on the medial side of the superior aspect at the femur’s greater trochanter [1]. Regarding its function, it is an external rotator of the hip when the hip is extended and a hip abductor when the hip is flexed [2]. The anatomical relationship between the piriformis muscle and other vasculonervous structures (e.g., the sciatic nerve, the superior gluteal artery, the superior gluteal nerve, the inferior gluteal artery and the inferior gluteal nerve) has been widely explored in the literature, and especially its relationship with the sciatic nerve. Although multiple anatomical variations are possible, the sciatic nerve generally exits the pelvic girdle caudally to the piriformis muscle (>90% of cases) [3]. In the remaining cases, most anatomical variations (with the sciatic nerve running through or above the piriformis muscle) occur among East Asians [4].

As a result of this close relationship at the exit of the greater sciatic notch, a sciatic nerve entrapment may occur. This clinical condition is known as piriformis syndrome [5,6]. Its incidence ranges from 0.3% to 36% and around 5% of the worldwide population is estimated to have piriformis syndrome [5,6]. Its clinical presentation consists of tingling, numbness or pain in the gluteal region that often expands down the back of the leg, and some mechanical demands (e.g., prolonged sitting, climbing stairs or stretching) can worsen the pain [5,6]. In addition, previous research has reported a considerable prevalence of active myofascial trigger points (MTrPs) located in the piriformis muscle in patients with radiating low back pain [7], patellofemoral syndrome [8] and chronic nonspecific low back pain [9].

Conventional therapies such as oral drugs or local infiltrations are often contraindicated in some populations due to potential complications [10]. For instance, skeletal muscle relaxants are effective agents for managing acute pain, yet their use raises safety concerns due to a high risk of adverse drug events. Over the past decade, the overuse of these medications, especially agents like carisoprodol (which was reclassified as a controlled substance by the DEA in 2012 due to its abuse potential and risk of dependency) has led to an increase in adverse effects, including sedation, headache, dizziness, blurred vision, nausea and vomiting. These medications, though commonly used for acute musculoskeletal back pain, are frequently taken in higher doses and for longer durations than recommended. Evidence supports their use only for acute pain episodes, highlighting the importance of their short-term use to prevent the masking of underlying conditions and their administration being based on individual factors including the duration and severity of symptoms, prior response to medications, potential side effects, desired benefits, comorbid conditions and costs. Alternatively, minimally invasive interventions such as dry needling are demonstrating promising results, with this treatment modality gaining increasing popularity [11,12,13,14,15]. Gattie et al. [16] recently reported that over half of the surveyed physiotherapists in the United States (55%) currently include dry needling in their practice, primarily in outpatient settings. Among those who use it, the procedure is commonly applied in three to six short sessions of less than 15 min and typically combined with other interventions like therapeutic exercise and joint mobilization.

Dry needling consists of intramuscular stimulation using thin filiform needles (normally between 0.20 and 0.32 mm of thickness) with a cone tip, without the use of any substance targeting the MTrPs identified previously by manual compression [17,18]. Systematic reviews and meta-analyses [19,20,21] support, with low- to moderate-quality evidence, the short-term effectiveness of dry needling (compared with sham dry needling, no interventions or other physical therapy treatment modalities) for pain and disability in musculoskeletal pain conditions. The underlying mechanisms explaining these effects have been hypothesized to be attributed to pain modulation (activating the body’s pain-inhibitory pathways and reducing pain transmission via central nervous system mechanisms), trigger point disruption (the needle’s insertion disrupts the MTrP’s taut band, releasing trapped chemicals that contribute to muscle pain), biochemical changes (needling decreases levels of pain-related chemicals like substance P and calcitonin gene-related peptide at the MTrPs, leading to pain relief) and local reflexes (the insertion can evoke a local twitch response, which helps reset muscle fiber tension, reducing sensitivity) [22]. In addition, dry needling seems to be an effective treatment specifically for managing patients with piriformis muscle syndrome. Tabatabaiee et al. [12] conducted a randomized clinical trial with 32 patients divided into two groups (dry needling versus ‘wait-and-see’), obtaining a significant pain intensity reduction for the dry needling group in the short term (7 days). Similarly, other case and case series reports [11,15], found US-guided dry needling to be effective for reducing pain intensity, drug intake and quality of life for at least 6 months.

While dry needling is a frequently chosen intervention for its efficacy in the short-term, there are many concerns about dry needling posed by physical therapists due to the risks of adverse events and reports of inadequate training [16]. Although most of the most common adverse events (occurring in approximately 39.6% of treatments) are minor (e.g., bleeding 16%, bruising 7.7% and pain during the intervention 5.9%) and these occur at a rate comparable to or lower than those seen with interventions like thrust joint manipulation or pharmacological treatments with opioids (where minor side effects can occur in up to 60.9% of cases [16]), major complications (such as pneumothorax, symptom aggravation, fainting or nerve injury), even if they occur rarely, should not be ignored [23,24,25].

Even if the universal precautions described for invasive procedures are followed (e.g., the use of gloves and correct skin disinfection as an infection protection measure for health care workers and patients, respectively), there are risks which cannot be prevented by following these practices [24]. Due to the anatomical proximity of the piriformis muscle to the sciatic nerve, and considering the existence of reports describing nerve injuries after dry needling [26,27], additional precautions for avoiding neuropraxia and other complications are needed.

Ultrasound imaging (US) is the most cost-effective supportive tool used by physiotherapists for guiding needles during invasive procedures. It should be noted that clinicians do not use US for detecting or guiding the needle to MTrPs because evidence is controversial regarding the utility of US in identifying myofascial trigger points [28,29] and elasticity imaging software significantly increases the price of US devices. Instead, their B-mode can be used for identifying the muscle targeted while avoiding the accidental puncture of high-risk structures such as nerves, vessels and viscera.

The rationale for conducting this research is justified by two reasons. First, there are many situations where US is not readily accessible and there is a need for testing whether blinded interventions are precise/safe or not. This is reflected in the literature, as some authors believe that palpation-guided interventions are safe and precise based on cadaveric studies [30,31,32], while in other regions the high risk of serious adverse events discourages this practice [33,34]. Regarding the piriformis muscle, a previous cadaveric study estimated that dry needling in the piriformis medial region results in an accidental puncture of the sciatic nerves in 11 out of 14 trials (78.5%) [35]. Secondly, given the potential for serious adverse events during dry needling in this location, developing predictive models to assess the safety of palpation-guided interventions in high-risk areas is crucial. If these models demonstrate high accuracy, physiotherapists would be able to perform palpation-guided interventions safely, thereby enhancing the overall safety of invasive procedures, by calculating the optimal needle length accurately. Conversely, if the models do not achieve acceptable accuracy, there will be evidence that dry needling should only be performed with ultrasound guidance, significantly reducing the risk of adverse events. Therefore, in both scenarios this approach not only promotes patient safety but also encourages further research into the efficacy of needling interventions in sensitive locations, prioritizing the patients’ safety.

Therefore, considering the successful results in previous studies aimed at developing prediction models to avoid adverse events during dry needling in other regions such as the rhomboid major, sartorius and soleus muscles [36,37,38], this study aimed to develop a predictive model based on easy-to-measure anthropometric and demographic variables to calculate the maximum needle length recommended for needling the piriformis muscle in the medial region (which is the most risky area to needle) without puncturing the sciatic nerve.

## 2. Methods

### 2.1. Study Design

A cross-sectional observational study was conducted to identify potential predictors of the deep piriformis muscle’s fascia depth (immediately superficial to the sciatic nerve) by calculating the correlation between the target and demographic and anthropometric data and, subsequently, calculating a linear regression model for assisting clinicians in the selection of optimal needle length during non-US-guided needling interventions. This report followed the recommendations of the Strengthening the Reporting of Observational studies in Epidemiology (STROBE) [39] guidelines and the checklist for cross-sectional studies, and all procedures were supervised and approved by a Local Ethics Committee, which ensured the participants’ rights were upheld in accordance with the Declaration of Helsinki.

### 2.2. Participants

Potential volunteers were screened for eligibility in a private University and its associated Hospitals, located in Madrid (Spain), during January 2022–April 2022. Recruitment efforts included local announcements and flyers distributed throughout the University and Hospitals, which encouraged individuals to inquire about participation in the study.

To be included in the study, volunteers had to (1) be between 20 and 60 years old. This age range was selected since the peak prevalence of piriformis syndrome is estimated to be at 30–50 years old [40]. In addition, participants had to (2) report the presence of at least one active MTrP (examined and classified following the Delphi study for examining MTrPs [16]) and (3) a positive response (considered a partial or total provocation of symptoms recognized by the patients as “familiar”) to the Freiberg maneuver (positioning the patient in the recumbent supine position, the examiner performs an internal rotation and adduction of the affected lower limb, with the hip flexed 30–45° and the knee totally extended), as this is demonstrated to be a recommended diagnostic criterion for patients with piriformis muscle syndrome [41].

Participants were excluded if they reported (1) that they were under any treatment which could affect their muscle tone (e.g., pharmacological or physiotherapy treatments), (2) relevant lower limb asymmetries, (3) a prior history of surgery in the lower limbs or lumbopelvic structures, (4) neuromuscular conditions compromising a normal lumbopelvic morphology (e.g., sarcopenia, myasthenia gravis, lateral amyotrophic sclerosis or spinal muscular atrophy) or (5) any other medical condition (e.g., tumor or fractures). Signing a written informed consent form was mandatory for all participants before their enrollment in the study.

### 2.3. Sample Size Calculation

Following the recommendations provided by Beneziuk et al. of including 10 to 15 participants per potential predictor [42] and considering a maximum of 5 predictors for avoiding an overestimated accuracy [43], a minimum sample size of 50 participants was required.

### 2.4. Demographic and Anthropometric Data

The demographic data collected included age (years), gender (male/female), height (m), weight (kg) and body mass index (BMI = weight/height^2^ [44]).

**Figure 1:**
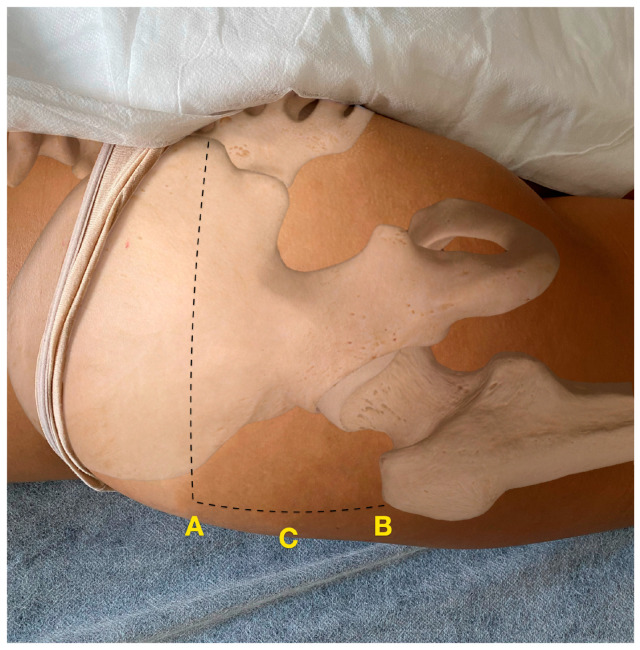
Hip circumference measurement location (C) at the midpoint between the posterior superior iliac spine projection, as the upper limit, (A) and the upper limit of the greater trochanter, as the lower limit (B).

The anthropometric indicator was the perimeter (in cm) at the midpoint between the posterior superior iliac spine (upper limit) and the upper limit of the greater trochanter (lower limit) (Figure 1).

**Figure 2:**
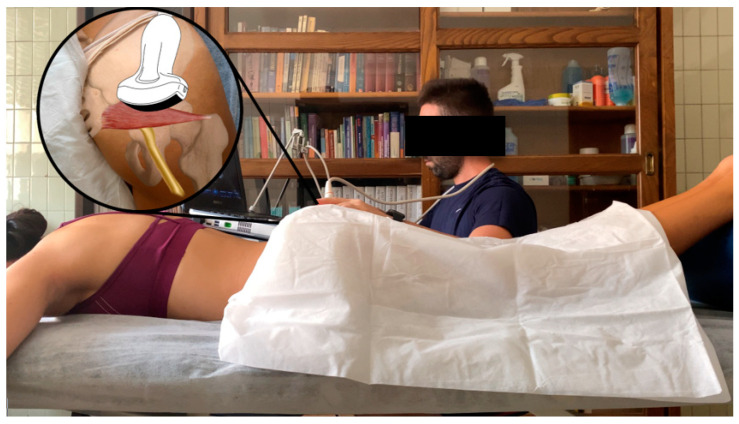
Ultrasound imaging acquisition: participants’ position and probe positioning.

Participants were placed in the prone position on a table, with hips in neutral position, knees extended, and a pillow placed under the feet, as shown in Figure 2. This position was selected since it corresponds to the greater sciatic notch and the piriformis muscle’s locations [1,2,3].

### 2.5. Ultrasound Imaging Procedure: Piriformis Deep Limit Calculation

Participants undergoing the US exam were placed in the same position described for collecting the anthropometric data. A single examiner with +10 years of experience in musculoskeletal US conducted all the measurements using a Mindray M8 device (Mindray Medical España S.L., Madrid, Spain) with a convex transducer C5-1s. Prior to each examination, the ultrasound device was calibrated to ensure high-quality imaging by adjusting several key settings. The frequency was selected based on the depth of the piriformis muscle and sciatic nerve, with higher frequencies used when they were identified to be more superficial, and lower frequencies applied at greater depths to ensure adequate penetration. The depth setting was adjusted to encompass both the piriformis muscle and the sciatic nerve while minimizing unnecessary surrounding tissue. The focus was positioned at the level of the sciatic nerve to enhance image clarity, as proper focus adjustment improves the resolution of the desired structures. Additionally, the dynamic range setting was optimized to enhance image contrast; a wider dynamic range was selected for the effective visualization of soft tissue structures, while a narrower range was used for increased contrast in areas with significant differences in echogenicity. Light pressure was applied during the examination to avoid compressing the subcutaneous tissue, which could lead to measurement bias.

The procedure followed for obtaining the piriformis muscle and sciatic nerve image was described by Siahaan et al. [45]. The authors conducted a diagnostic accuracy test to determine the sensitivity, specificity and best cut-off point for measuring ultrasound-guided piriformis muscle thickness in piriformis syndrome, demonstrating that 0.995 cm is the optimal cut-off point for diagnosing piriformis syndrome (94.8% sensitivity and 87.9% specificity). First, the transducer was placed using surface landmarks (from the midpoint between the posterior superior iliac spine and the sacral hiatus to the greater trochanter) and glided caudally to visualize a long-axis view of the piriformis muscle.

**Figure 3:**
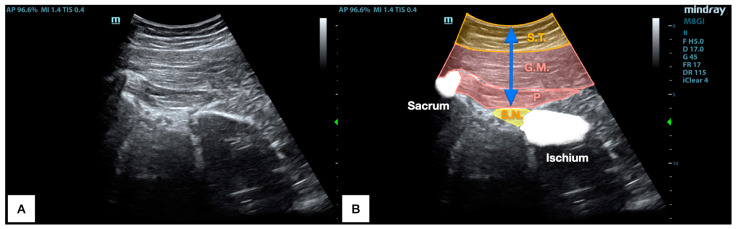
Raw ultrasound image acquired (**A**) and labeled image, highlighting the main structures (**B**). Blue arrow: distance between the skin and the sciatic nerve; G.M.: gluteus maximus; P: piriformis muscle; S.T.: subcutaneous tissue.

As shown in Figure 3, US allows for the visualization of the piriformis muscle thickness (between the gluteus maximus and the sciatic nerve) and osseus references (sacrum lateral border and ischium). Finally, a perpendicular caliper measured the distance between the skin and the deeper fascia of the piriformis muscle (cm).

### 2.6. Statistical Analysis

Statistical analyses were run on SPSS v.27 (Armonk, NY, USA) for Mac OS. After verifying a normal distribution of the data with Saphiro–Wilk tests and histograms (normality was assumed for *p* values > 0.05), descriptive analyses reported the central tendency and dispersion of continuous variables and the frequency and percentage of categorical variables. Student’s *t*-tests for independent samples were used for assessing gender differences.

A Pearson’s correlation matrix was calculated for two primary purposes. First, it was used to identify the strength and direction of the associations among the variables, where positive *r* values indicated directly proportional associations and negative values indicated inversely proportional associations. The absolute values of *r* ranged from 0 (interpreted as an absolute absence of correlation) to 1 (indicating a perfect association). Second, the correlation matrix helped identify multicollinearity and shared variance among the variables, which is crucial in regression analyses. Multicollinearity refers to a situation where two or more independent variables are highly correlated, leading to redundancy in the model and potentially skewing the results. By setting a threshold of *r* > 0.80, the matrix can detect such relationships, allowing the exclusion of variables that exhibit shared variance. This exclusion is essential to minimize bias and prevent the overestimation of accuracy in the regression model, ensuring that each predictor’s contribution is assessed independently and accurately [46].

Following this analysis, only the variables that showed statistically significant correlations (*p* < 0.05) and no shared variance with the deeper piriformis muscle fascia depth were included in a forward stepwise multiple linear regression model. The significance criterion for inclusion in the regression equation was set at *p* < 0.05. Adjusted changes in R^2^ were reported for each step to determine the individual variance contribution of each predictor [43].

## 3. Results

**Figure 4:**
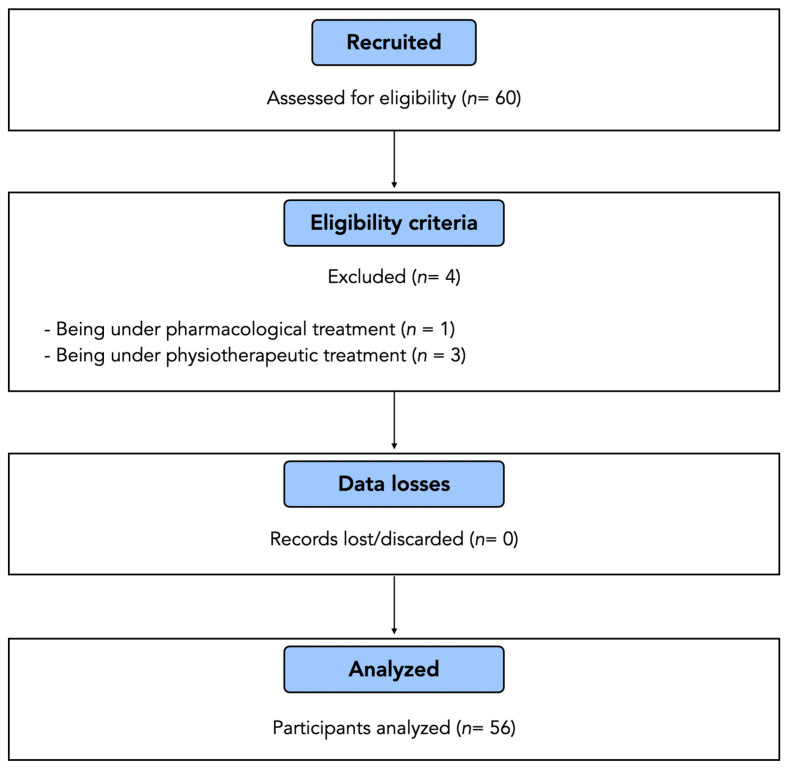
Participants’ flow chart: recruitment process, application of eligibility criteria, data losses and number of participants analyzed.

Although 60 subjects were initially recruited, four participants were excluded as they were under pharmacological (*n* = 1) or physiotherapy (*n* = 3) treatment. The remaining 56 participants were finally included and analyzed, with no data losses. The participants’ flow diagram is illustrated in Figure 4.

The sociodemographic data of the sample and the comparison between males and females are available in Table 1. In summary, males and females had comparable ages and BMIs (*p* > 0.05), but males were significantly taller (*p* < 0.001), heavier (*p* < 0.001) and had a greater hip perimeter than females (*p* = 0.007). Regarding the distance between the skin and the piriformis deeper fascia, no significant differences were found between males and females (*p* > 0.05).

Table 2 contains the Pearson’s correlation matrix data regarding the associations found among the anthropometric, demographic and ultrasonographic data. Weight, BMI and hip perimeter were shown to be significantly associated with the piriformis deeper fascia depth (all, *p* < 0.01), while age and height were not associated with it (*p* > 0.05).

Finally, Table 3 summarizes the regression analyses used to predict the piriformis deeper fascia depth. Since multicollinearity and shared variance were found among the three potential predictors associated with piriformis deeper fascia depth, only the hip perimeter was included in the regression model (as it showed the greatest association). The hip perimeter explained 37.9% of the piriformis muscle’s deeper fascia depth.

**Figure 5:**
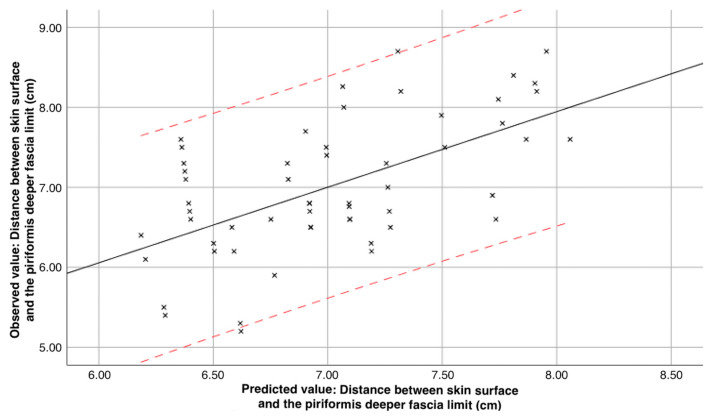
Scatter plot illustrating the relationship between observed and predicted values for the distance between the skin surface and the piriformis deeper fascia limit (crossmarks, in cm). The solid black line represents the linear regression fit, while the dashed red lines indicate the upper and lower 95% prediction intervals. The regression equation is given by y=0.39+0.95×x.

The plot illustrated in Figure 5 shows the observed depth of the piriformis for each participant (Y-axis) and the predicted value calculated based on this regression model (X-axis). Predicted values in a regression model are calculated using the estimated regression equation, which expresses the relationship between the dependent variable and one or more independent variables (y=0.39+0.95×x, where y is the predicted value, b_0_ = 0.39 is the intercept, b_1_ = 0.95 is the coefficient of the independent variable and x represents the actual value of the independent variable). To calculate the predicted value for any given observation, the actual value of x is substituted into the equation. The intercept is added to the product of the coefficient and the independent variable. This process generates a predicted value for the dependent variable, which reflects the expected outcome based on the specified input.

## 4. Discussion

This is the first study conducted to assist clinicians during the dry needling of the piriformis muscle on the selection of the optimal needle length to avoid the accidental puncture of the sciatic nerve. Although the obtained results demonstrated that there were multiple easy-to-measure factors associated with the piriformis deep fascia limit (immediately superficial to the sciatic nerve), including weight, body mass index and hip perimeter, hip perimeter emerged as the best predictor for determining the appropriate needle length during the dry needling of the piriformis muscle due to its strong association with the anatomical depth of the piriformis muscle’s deep fascia relative to the sciatic nerve. Unlike weight and BMI, which provide a more generalized assessment of body size and composition, hip perimeter directly reflects the anatomical breadth of the gluteal region where the piriformis and sciatic nerve are located. This specific measure captures the variability in soft tissue thickness, muscle bulk, and overall pelvic structure in the area targeted by clinicians during dry needling.

Although US is the most cost-effective tool for ensuring the patients’ safety during dry needling interventions and the integration of point-of-care US in musculoskeletal physiotherapy offers substantial clinical benefits, it still presents notable challenges in its technical execution, education, and governance, as summarized in a recent article which proposed a framework for point-of-care musculoskeletal US and US-image-guided interventions performed by physiotherapists [47]. Physiotherapists using US can enhance patient assessments by visualizing anatomical structures in real time, but this advantage requires high-level image acquisition and interpretation skills, competencies typically gained through extensive training and mentorship. The task of differentiating normal anatomical variations from pathological findings demands precise probe handling, image optimization, and in-depth anatomical knowledge, which can be difficult to achieve and maintain for clinicians without a formal background in imaging. Additionally, the musculoskeletal field is characterized by diverse patient conditions and complex anatomical regions, making it challenging to establish a standardized scope of practice for physiotherapists using US. Professional training resources, especially for advanced imaging techniques, are often limited, and there is a need for formal competency assessments to ensure their safe and accurate use. Furthermore, governance issues arise due to varying regulatory guidelines, particularly concerning the validation of skills and legal permissions for conducting imaging and guided interventions. The absence of unified standards can leave physiotherapists vulnerable to legal and professional risks, especially in cases of misdiagnosis. To address these challenges, structured education, comprehensive clinical training and a robust governance framework are essential for the safe and effective integration of PoCUS in physiotherapy practice.

While the Introduction provided a general overview of the adverse events associated with palpation-guided dry needling to justify the need for strategies aimed at improving patient safety and avoiding the accidental puncture of important structures [23,24,25], a literature search was also conducted to analyze the factors associated with the occurrence of these events, particularly focusing on post-needling nerve injuries, which are among the most serious adverse events in this context. Severe or major adverse events have been reported at rates between 0.01% and 0.87% per treatment [23,48], accounting for approximately 13% of all adverse events [49]. Throughout a physiotherapist’s career, major adverse events occur in ranges from 0% to 15% of cases. While syncope and symptom exacerbation are the most reported (15%), nerve injuries occur at a rate of 0.7% [16]. Furthermore, certain factors were found to be significantly associated with the reporting of major adverse events during dry needling procedures. Specifically, being male, having more than four years of experience in dry needling, undergoing more than 60 h of formal training and possessing a high self-rated skill level were all associated with an increased likelihood of reporting major adverse events. Other factors such as verbal suggestion seem to have no impact [50].

One warning sign commonly used to detect nerve puncture is an electric shock sensation, defined by the patients as an intense sourness, numbness, heaviness, or distension feeling [51]. Although this electric shock is wanted in acupuncture (as it seems to play a determinant role in the treatment’s success and efficacy) [52], other researchers have suggested that the treatment should be interrupted immediately to avoid the nerve injuries described in the Introduction [23,24]. One possible reason explaining why this evidence is not conclusive for determining whether this warning sign is a normal needling sensation or not could be the similarity between the description of this sign and the puncture response of active MTrPs (which reproduce the patients’ symptomatology, including referred pain to the limbs). Therefore, differentiating between a normal MTrP puncture and a nerve puncture could be challenging.

Although Guo et al. found no basic experimental studies describing the consequences of repeated nerve puncture during needle manipulation (which normally consists of movements such as lifting, twisting and twirling), the authors described that the myelin sheath, axons and both the extra and intraneural vascular systems are likely to be punctured when needles stab the nerve. The resulting hematoma can squeeze the surrounding nerve tissues and trigger classic nervous inflammation processes and neuropathic inflammation [51].

In order to avoid nerve damage during this procedure, clinicians and researchers should follow two key strategies for increasing the procedure’s safety. The first is using the landmarks described for accurately targeting the piriformis muscle. Kearns et al. [53] approached the most complex area by introducing a needle immediately lateral to the most lateral border of the sacrum and angling the needle perpendicular to the contour of the posterior pelvis toward the symphysis pubis. This procedure resulted in 100% accuracy in reaching the piriformis muscle [54]. Since the authors justified their needle selection by a pragmatic decision, setting a maximum limit of 10 cm (which can lead to accidental nerve puncture), the second key message is to consider the hip circumference at the location described in this study. Although the explained variance was a 37.9%, this simple calculation provides a more accurate approximation for avoiding nerve puncture.

### 4.1. Limitations

Although this report could assist clinicians and researchers in creating a safer practice, some caution should be advised. The most important limitation is that 37.9% of the variance being explained is not enough to ensure risk-free interventions. This study included a sample with limited characteristics and further studies including a wider dispersion of sociodemographic and anthropometric characteristics and other participant profiles (including asymptomatic subjects, other clinical entities or subjects with anatomical variations) may improve the accuracy of the model and lead to more generalizable conclusions. In addition, the sample assessed in this study showed similar anatomical characteristics (100% of participants had a single nervous trunk deep to the piriformis muscle), limiting the generalizability of the model to populations with different anatomical characteristics. However, multiple anatomical variations are described and the only method used to ensure a safe procedure is US. Finally, the reliability of physical examination in determining the presence of MTrPs is considered poor, since physical examinations and pressure pain threshold measurements rely significantly on the clinician’s sensitivity, judgment and expertise, as well as the patients’ pain reports [55]. Therefore, even if we combined a provocation test with an MTrP physical examination, this subjectivity may impact the sample’s characterization.

### 4.2. Future Directions

A clear understanding of the risks and adverse effects associated with dry needling is essential, along with the factors that influence their frequency and severity, including therapist experience, anatomical location and needle type. Future research should prioritize original studies and systematic literature reviews to explore these questions. In addition, calculating further regression models is essential for determining which procedures can be safely performed using palpation references and which require ultrasound guidance. Research should focus especially on high-risk areas, such as the thoracic and cervical regions, and locations with critical anatomical relationships to vascular and nerve bundles.

## 5. Conclusions

This study found that the hip circumference accounted for only 37.9% of the variance in the depth of the piriformis deep fascia at the most complex anatomical location in patients with piriformis muscle syndrome. This limited accuracy does not support the use of palpation-guided interventions in this area. While anatomical landmarks may provide reasonable accuracy for reaching the piriformis muscle and the hip circumference partially explains the depth of the sciatic nerve, these methods are insufficient to reliably avoid an accidental puncture of the sciatic nerve. Therefore, only ultrasound-guided interventions are recommended for this location, as it is the only method that ensures a completely safe procedure.

## Figures and Tables

**Table 1:** Sociodemographic and anthropometric characteristics of the sample.

Variables	Sample (*n* = 56)	Gender		
		Males (*n* = 34)	Females (*n* = 22)	Difference
Age (years)	31.2 ± 5.1	31.5 ± 6.3	30.8 ± 9.6	0.7 (−3.5; 5.0) *p* = 0.732
Height (m)	1.74 ± 0.07	1.79 ± 0.08	1.66 ± 0.04	0.13 (0.07; 0.18) *p* < 0.001
Weight (kg)	68.4 ± 11.3	74.5 ± 11.2	59.0 ± 3.0	15.5 (8.4; 22.7) *p* < 0.001
Body mass index (kg/m^2^)	22.4 ± 2.5	23.1 ± 2.4	21.5 ± 1.9	1.6 (0.1; 3.5) *p* = 0.051
Hip perimeter (cm)	94.0 ± 6.2	96.5 ± 6.0	90.2 ± 4.7	6.2 (1.8; 10.6) *p* = 0.007
Piriformis deeper fascia depth (cm)	7.0 ± 1.3	7.1 ± 0.8	6.9 ± 0.9	0.2 (−0.2; 0.7) *p* = 0.298

**Table 2:** Pearson product moment correlation matrix.

	1	2	3	4	5
1. Age					
2. Height	0.003				
3. Weight	0.236	0.789 **			
4. Body Mass Index	0.384 **	0.243	0.785 **		
5. Hip perimeter	0.264 *	0.564 **	0.866 **	0.811 **	
6. Piriformis deeper fascia depth	−0.053	0.252	0.550 **	0.614 **	0.624 **

* *p* < 0.05; ** *p* < 0.01.

**Table 3:** Summary of the regression analyses used to determine predictors of piriformis deeper fascia depth.

	Predictor Outcome	Adj R^2^	B	SE B	95% CI	β	t	*p* Value
Piriformis Deeper Fascia Depth	Step 1 Hip perimeter	0.379	0.085	0.014	0.056; 0.114	0.624	5.875	<0.001

## Data Availability

The raw data supporting the conclusions of this article will be made available by the authors on request.
